# A Statistical Framework for Improving Genomic Annotations of Prokaryotic Essential Genes

**DOI:** 10.1371/journal.pone.0058178

**Published:** 2013-03-08

**Authors:** Jingyuan Deng, Shengchang Su, Xiaodong Lin, Daniel J. Hassett, Long Jason Lu

**Affiliations:** 1 Division of Biomedical Informatics, Cincinnati Children’s Hospital Medical Center, Cincinnati, Ohio, United States of America; 2 Department of Environmental Health, University of Cincinnati, Cincinnati, Ohio, United States of America; 3 Department of Biochemistry and Molecular Genetics, University of Cincinnati, Cincinnati, Ohio, United States of America; 4 Department of Computer Science, University of Cincinnati, Cincinnati, Ohio, United States of America; 5 Department of Management Science and Information Systems, Rutgers University, Piscataway, New Jersey, United States of America; University of Toronto, Canada

## Abstract

Large-scale systematic analysis of gene essentiality is an important step closer toward unraveling the complex relationship between genotypes and phenotypes. Such analysis cannot be accomplished without unbiased and accurate annotations of essential genes. In current genomic databases, most of the essential gene annotations are derived from whole-genome transposon mutagenesis (TM), the most frequently used experimental approach for determining essential genes in microorganisms under defined conditions. However, there are substantial systematic biases associated with TM experiments. In this study, we developed a novel Poisson model–based statistical framework to simulate the TM insertion process and subsequently correct the experimental biases. We first quantitatively assessed the effects of major factors that potentially influence the accuracy of TM and subsequently incorporated relevant factors into the framework. Through iteratively optimizing parameters, we inferred the actual insertion events occurred and described each gene’s essentiality on probability measure. Evaluated by the definite mapping of essential gene profile in *Escherichia coli*, our model significantly improved the accuracy of original TM datasets, resulting in more accurate annotations of essential genes. Our method also showed encouraging results in improving subsaturation level TM datasets. To test our model’s broad applicability to other bacteria, we applied it to *Pseudomonas aeruginosa PAO1* and *Francisella tularensis novicida* TM datasets. We validated our predictions by literature as well as allelic exchange experiments in *PAO1*. Our model was correct on six of the seven tested genes. Remarkably, among all three cases that our predictions contradicted the TM assignments, experimental validations supported our predictions. In summary, our method will be a promising tool in improving genomic annotations of essential genes and enabling large-scale explorations of gene essentiality. Our contribution is timely considering the rapidly increasing essential gene sets. A Webserver has been set up to provide convenient access to this tool. All results and source codes are available for download upon publication at http://research.cchmc.org/essentialgene/.

## Introduction

Large-scale systematic analysis of gene essentiality is an important step closer toward unraveling the complex relationship between genotypes and phenotypes [1]. However, such analysis cannot be accomplished without unbiased and accurate annotations of essential genes.

Whole-genome knockout experiments produce the most accurate essential gene annotations, but they often take a consortium of labs many years to complete in even one organism. It is not surprising that single-gene knockout results are currently only available in a handful of well-studied model organisms, such as *Escherichia coli* [2] and *Saccharomyces cerevisiae* [3]. Computational methods for predicting essential genes, e.g., homology mapping [4], [5], [6], constraint-based methods [7], [8], [9], [10] and supervised learning [11], [12], [13], [14], [15], are often useful in reducing the cost and labor. However, they have limited applicability to understudied species. For example, supervised learning depends on a partial list of known essential genes which is often unavailable for understudied species. Constraint-based methods are often limited to metabolic and signaling pathways and require prior knowledge of pathways. Homology mapping works best when the model and target organisms are closely related. Otherwise, the prediction coverage is often low and it ignores the unique physiology of the subject species as people have found that orthologs do not necessarily have the same degree of essentiality [16].

Therefore, to interrogate the essential genes in understudied species, whole-genome transposon mutagenesis (TM) followed by sequence-based identification of insertion sites is often the most practical and the most frequently used experimental approach [17]. Using this approach, essential genes for a variety of understudied bacterial species, such as *Pseudomonas aeruginosa* and *Francisella tularensis novicida*, have been identified, greatly increasing the insights into the essential processes necessary for growth of these bacteria under defined conditions. As of May 2012, we have found at least 24 genomic scale TM studies in 19 bacterial species from literature (**Table S1**), all of which except two were generated within the past ten years. For the past three years, we have seen at least nine such new datasets from different organisms. As researchers are starting to analyze conditional essential genes, that is, essential genes under virtually unlimited growth conditions, we expect the number of available TM datasets will rapidly increase in the future years.

As a result of this rapid increase, in current genomic databases, e.g., [18], [19], [20], many of the annotations of microbial essential genes are derived directly or indirectly from TM experiments. However, unlike single-gene knockouts, TM has intrinsic biases (see below). Without correcting these biases, the TM assignments will contain hundreds of mis-annotated essential and non-essential genes in each organism, which has caused substantial confusion among the genetics community.

Transposons are segments of DNA that can move (transpose) from one location in a genome to another [21], [22]. The locations in which a transposon can move depends on the sequence that the transposase recognizes and cleaves, although the recognition sequence for some transposons is unclear or has yet to be determined. TM results in disruption of the region of the genome where the transposon is inserted. If an insertion within a predicted ORF allows the resulting strain to form a colony on appropriate solidified media, it is unlikely that ORF is essential for viability under those conditions (**Fig. S1**). Therefore, TM identifies essential genes using a “negative” approach, i.e., identifying many regions that are not essential and presuming that everything else is essential.

Due to the random nature of transposon insertion events, there are a number of factors that may create systematic biases in TM experiments. For example, it is inevitable that some genes, especially shorter ones, will be missed simply by chance [23], [24], [25]. This will create false positive errors in which non-essential genes are determined as essential by TM. On the other hand, the insertion may take place at any part of a gene, such as the extreme ends, which may not fully disrupt the function of the gene product [23], [24], [25], [26], [27], [28], [29], [30]. This will create false negative errors in which essential genes are determined as non-essential by TM. These biases from TM experiments have introduced substantial errors in essential gene annotations in current genomic databases [19]. In order to render the large-scale integrative and comparative analysis of essential genes possible, these biases must be quantitatively assessed and corrected.

Several models were previously developed to tackle the biases in TM. For example, Lamichhane et al. used a Bayesian framework to estimate essential genes in *Mycobacterium tuberculosis* with a subsaturation level of TM [28], [31]. Jacobs et al. used a neutral base pair model to reach an estimation of 307 essential genes in *P. aeruginosa PAO1* with a saturation level of TM [29]. However, the key limitation of the existing models is that they only take into account the insertions in viable mutants and ignore those that disrupt essential genes because these mutants die and, as such, are not observable. Therefore, the number of real insertions is underestimated to equal the observed insertions. Also, most of the existing models assume a gene’s probability of being inserted only depends on its length and one insertion is sufficient to disrupt a gene’s function. However, the TM results often show “hot” and “cold” spots in the genome for transposon insertions. Furthermore, it may often require multiple insertions to disrupt a gene’s functions, depending on the site of the insertion.

Given these caveats, we developed a novel Poisson model based statistical framework to simulate the TM insertion process and subsequently correct the experimental biases. Briefly, the statistical framework works as follows: We first quantitatively assessed the effects of potential factors that may affect the accuracy of TM results, such as gene length and relative insertion positions, and subsequently incorporated relevant factors into the framework. Through iteratively optimizing parameters, we finalized the model and inferred the actual insertion events occurred in each gene given the observed insertion information. Finally we described each gene’s essentiality on probability measure, and provided corrections towards possible biases in the TM assigned annotations.

We took advantage of the definitive mapping of essential genes in *E. coli* MG1655 strains determined by single gene knockout experiments (PEC set) [2] to identify the errors in the essential gene annotations produced by TM experiments (Gerdes set) [23] by comparing their assignments. Although the single-gene knockout experiments are not completely error free, they have been considered the least error-prone [2]. We also realized that the essential genes uniquely identified by TM may have biological significance as they may represent genes essential for fitness as suggested by Gerdes et al. [32]; however, since our focus was on those essential for survival, we still referred to them as “errors”. Also note, our model is not dependent on the single-gene knockout results in the target organism (see the **Discussion** section). The PEC dataset was only used for assessing the errors in TM dataset and evaluating our model’s performance.

## Results

### 1. Assessing Overall Error Rates in the TM Annotations of Essential Genes

Using the PEC set as a reference, we assessed the overall error rates in the Gerdes dataset. We examined the subset of genes appearing in both datasets. This subset contains 3833 genes in total, including 615 TM-assigned essential genes (TmEs) and 3218 TM-assigned non-essential genes (TmNs), and covers 90% (3833 out of 4291) of all genes in the TM dataset.

According to the intersection with the PEC set, the Gerdes dataset can be divided into four mutually exclusive fractions (**Table 1**): true essential by TM (TETmE, essential assigned by TM and PEC), false essential by TM (FETmE; essential assigned by TM but non-essential in PEC), false non-essential by TM (FNTmN; non-essential by TM but essential in PEC) and true non-essential by TM (TNTmN; non-essential by both TM and PEC). In this report, we defined the TM essential error rate (TmER) as the proportion of the FETmEs over the total TmEs; likewise, the TM non-essential error rate (TmNR) was defined as the proportion of the FNTmNs over the total TmNs. Among the 615 TmEs, 186 were TETmEs, yielding a TmER of 70%. Similarly, the TmNR was 2.3%. Typically, in TM experiments the TmNR is low, but the TmER is relatively high.

**Table 1:** Using the PEC dataset as gold-standards to identify the false essential and non-essential genes in the TM dataset.

*E – Essential		PEC dataset (Gold Standard)	
*N – Non-essential		E (259)	N (3574)
**TM dataset**	TmEs (615)	186 (TETmEs)	429 (FETmEs)
**(Gerdes set)**	TmNs (3218)	73 (FNTmNs)	3145 (TNTmNs)

### 2. Assessing the Effects of Four Factors on the Accuracy of TM Experiments

**Figure 1:**
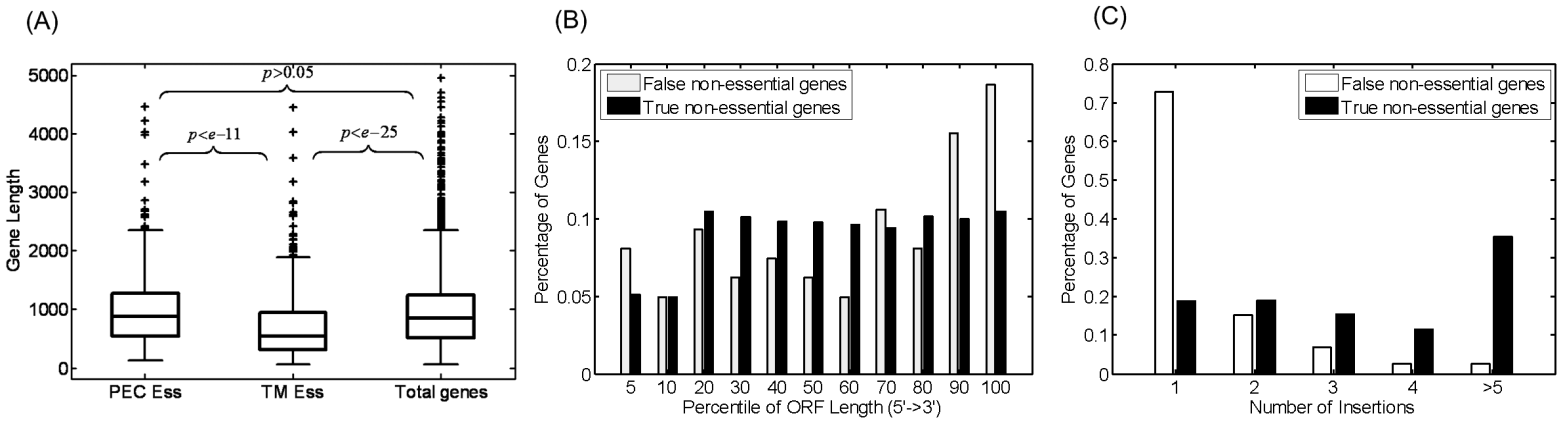
Three factors have strong associations with false TM assignments. (A) Gene length. The lengths of TmEs are significantly shorter than those in the PEC dataset and total genes. Many of these short genes may be false essential genes. (B) Position of insertions. Essential genes mistakenly assigned to be non-essential by TM often have insertions in the 25% extreme-ends (5% in 5′ end and 20% in 3′ end). These insertions do not completely disrupt a gene’s function. (C) Number of insertions. 75% of the essential genes mistakenly assigned to be non-essential by TM only have one insertion in them.

Previous studies suggested the accuracy of TM experiments may be affected by four main factors: gene length, insertions in the distal regions, number of insertions per gene and polar effects [23], [29]. To quantitatively assess each factor’s influence on the accuracy of TM results, we examined the association of each factor with the FETmEs and FNTmNs, respectively (**Fig. 1**).

#### (1) Gene length: potentially causing false positive errors

In TM experiments, the genes that have never been detected with transposon hits will be assigned as essential (**Fig. S1**). The average detectable insertion density is about 1 per 400 bp in TM experiments under saturation levels [23], [24], [25]. This suggests relatively short genes (e.g. ≤300 bp) would easily be missed simply by chance, and thus will be incorrectly labeled as essential.

To quantitatively assess its influence, we compared the length of essential genes in the Gerdes and PEC sets, using the total genes as a control (**Fig. 1A**). The student t-test shows that the average length of essential genes in the Gerdes set (730 bp) is significantly shorter than that in the PEC set (1,003 bp) (P-value <1E-11). It is also significantly shorter than total genes (982 bp) (P-value <1E-25) while the difference between the PEC set and total genes is considered not significant (P-value >0.05).

#### (2) Insertions in the 5′- and 3′-ends of genes: potentially causing false negative errors

In TM experiments, sometimes insertions occurring at the extreme ends of a gene’s coding sequence may not sufficiently disrupt its function [23], [24], [25], [26], [27], [28], [29], [30]. In these cases, essential genes may be mistakenly assigned as non-essential genes, creating a false negative error. We compared the distributions of the position of insertions within the ORFs between FNTmNs and TNTmNs. As expected, the FNTmNs have a higher percentage of transposon insertions in the 3′- and 5′-ends than TNTmNs (**Fig. 1B**). To assess the significance of this difference, we simulated pure random insertion experiments within the coding sequences (see **Methods**). The P-values showed that in the 20%-most of the 3′-end and the 5%-most of the 5′-end regions, the FNTmNs have significantly more insertions than TNTmNs (**Table S2**). We named it the “25% extreme ends” rule and used it later in the model.

#### (3) Number of insertions per gene: potentially causing false negative errors

Occasionally, a few insertions in a gene are insufficient to completely disrupt its function, especially when the target gene is relatively long [23], [24], [25], [26], [29], [33]. We plotted the distribution of the number of insertions per gene for both FNTmNs and TNTmNs (**Fig. 1C**). The histogram showed that about 75% of the FNTmNs that were mistakenly assigned to be non-essential by TM only harbored a single insertion. The average insertion number per gene among FNTmNs was 1.56, significantly smaller than TNTmNs (4.13) (P-value ≤1E-26).

#### (4) Polar effects: potentially causing false positive errors

In TM experiments, polar effects can occur when a transposon inserts in a dispensable gene and prevents the transcription of its downstream essential genes in the same operon [23], [24], [25]. Because the insertion in this dispensable gene actually disrupts the entire downstream essential genes and causes death of these mutants, this dispensable gene will be incorrectly labeled as essential, causing a false positive error.

To detect the number of FETmEs caused by polar effects, we examined all TmEs within each of 2,665 operons that were inferred experimentally or computationally (see **Methods**). If there exists a TETmE that resides downstream of a FETmE, this FETmE is considered to be potentially caused by a polar effect. Among the 429 FETmEs, only 46 of them may be caused by polar effects. The small number is likely due to the fact that many TM experiments have been designed to prevent polar effects, typically by designing transposons with a strong or regulatable promoter downstream of the transposase but still within the transposon.

### 3. Developing the Statistical Framework to Correct TM Errors

**Figure 2:**
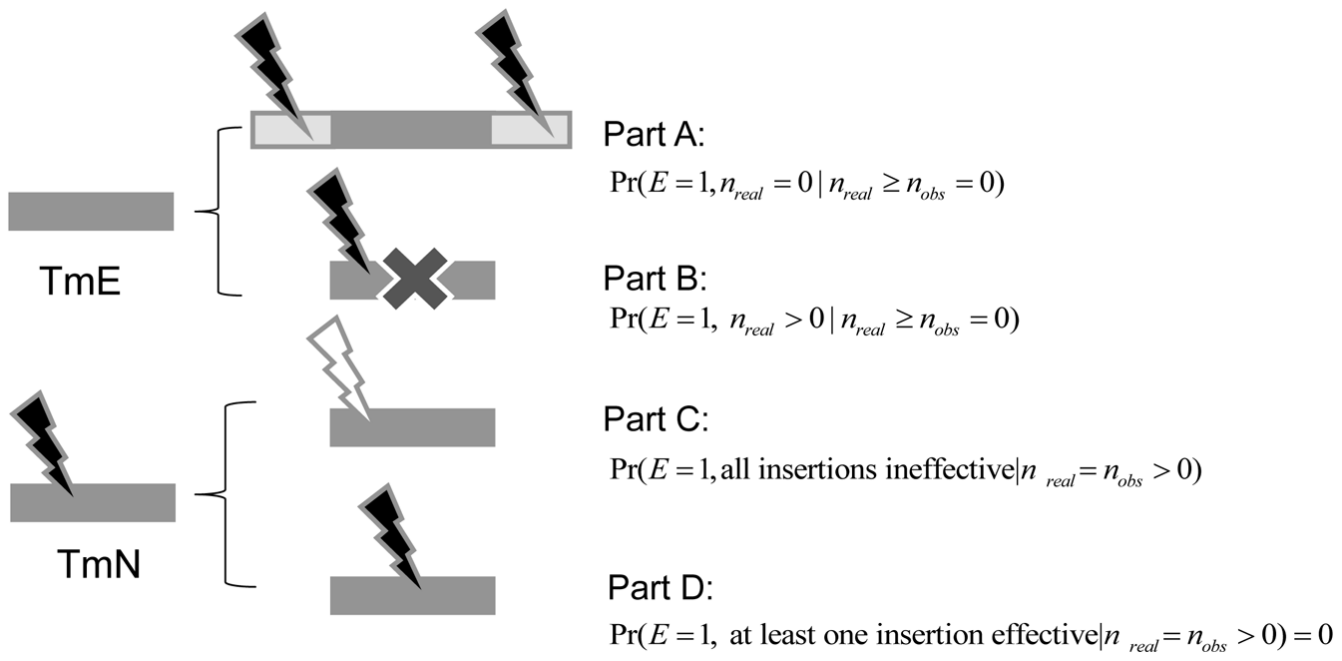
Illustration of the statistical model. In a TM experiment, if a gene has no observed insertions, meaning it is TM essential or TmEs, what could it be? There are two possibilities: (1) Part A: It never had any insertion and was missed by all transposons by chance. This means we do not have useful information to infer what this gene could be, and it is completely blind for us. For any blind gene, we can only try our best guess and assume that the chance of that gene to be essential 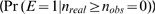 is equal to the overall essential gene rate (Pr(overall essential)), and that a gene to be non-essential is equal to 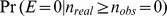 = 1-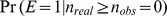. (2) Part B: It actually had insertions, but all inserted mutations died. 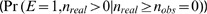 This means that this gene is truly essential. In this way, we can now split the TM assigned essential genes into two parts, TETmE and FETmE. Similarly, if in the TM experiment, a gene is observed to have insertions, meaning it is TM nonessential, what could it really be? There are also two possibilities: (1) Part C: All these observed insertions are ineffective, and did not interrupt the gene function. This means again we are blind about this gene. So it has a certain chance to be essential 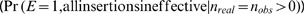, and also has a certain chance to be nonessential 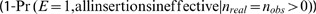. (2) Part D: There was at least one effective insertion, and it did interrupt the gene function. 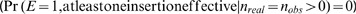. This means this gene is truly non-essential.

We then developed a statistical framework that is capable of incorporating potential factors that have strong associations with FETmE and FNTmN assignments. This model not only estimates the overall error rates, but also assigns a score to indicate the probability that an individual gene is essential given the TM assignments. We incorporated three of the above four potential factors into our model. Since polar effects are only responsible for a relatively few number of FETmEs, we chose not to include this factor into our model. The general idea of this model is illustrated in **Fig. 2**.

This model requires two assumptions:

- Transposons insert randomly and independently into the coding region of a gene; and
- Each transposon has the same ability to disrupt a gene’s function, although this ability may vary at different regions of a gene.

Assumption (A) does not require transposon insertions to be uniformly distributed along the entire genome because of the insertion “hot” and “cold” spots observed in microbial genomes and thus provides a more realistic approximation of the process of transposon insertions.

If we assume that transposons insert into a gene independently and the insertions occur at a constant rate during mutagenesis, then this process can be characterized by a Poisson distribution [34], [35]. The probability that there are 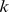 insertions occurring within a gene with length 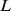 can be expressed as:
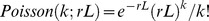
Here 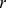 is the local insertion density on the DNA fragment, estimated by counting the number of insertions within a 30 kb-long region flanking the coding sequence. If *k* = 0, this equation describes the probability that this ORF is missed in TM experiments.

Based on assumption (B), we defined two parameters *P*_1_ and *P*_2_ both in the range of (0, 1) representing the probability that an individual transposon insertion disrupts a gene’s function when the insertion occurs at the 25% extreme ends or in the middle of a gene, respectively. We assumed the same probability (*P*_1_) for the 5%-most of 5′-end and 20%-most of 3′-end to disrupt a gene’s function.

Under these two assumptions, we can calculate the probability of being essential for each gene given the TM assignments.

- If it is a TM assigned essential gene (TmE), we have:
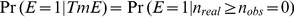
(1)
- Similarly, if it is a TM assigned non-essential gene (TmN), we have:
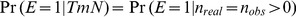
(2)

Here *E* is a binary variable and *E = *1 if this gene is essential; otherwise, it is non-essential. 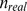 represents the real number of insertions occurring in this gene during the TM experiment and 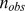 represents the observed number of insertions in the TM dataset. Here 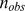 *= *0 if the gene is assigned as essential by TM, and 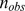
>0 if it is assigned as non-essential. 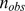 can be further separated into two parts 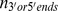 and 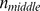 to represent the observed insertions occurring at the 25% extreme ends or in the middle of a gene, respectively. In the transposon insertion process, if an insertion hits a true essential gene and disrupts its function, the inserted mutant will die and this insertion will not be observable in the TM dataset; therefore the real insertion number 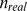 should be greater or equal to the observed insertion number 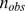. While for a non-essential gene, no matter whether an insertion disrupts its function or not, it will not die. Thus the real insertion number 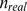 always equals to the observed insertion number 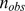.

After the derivation of equations, we then used an iterative procedure [36], [37], [38] to estimate the values of unknown parameters that determine Eqs. (1) and (2) (details see **Methods**).

### 4. Validating the Model in *E. coli* TM Dataset

The Gerdes dataset contains 615 TmEs and 3218 TmNs. Using the above algorithm, we found the converged *P*_1_ = 0.942, *P*_2_ = 0.984 and the overall essential rate 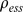 = 12.84%. Since our model assigned each individual gene a score to indicate its probability of being essential, we ranked these genes in the TmEs and TmNs separately. Among the 615 TmEs, the expected number of essential genes (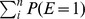) we estimated was 480. Using the expected number of essential genes as the cutoff, the top 480 genes were named predicted essential genes by our model among the TM-assigned essential genes (PETmEs) and the remaining 135 genes were predicted non-essential genes by our model among the TM-assigned essential genes (PNTmEs). Similarly, among the 3218 TmNs, the expected number of essential genes we estimated was 12. Using this cutoff, the top 12 genes were named predicted essential genes by our model among the TM-assigned non-essential genes (PETmNs) and the remaining ones were predicted non-essential genes among the TM-assigned non-essential genes (PNTmNs).

To assess the accuracy of our predictions, we compared our results with the PEC dataset (**Table 2**). Among the 480 PETmEs, 176 (or 37%) were true essential, significantly higher than that in the original TmEs (186/615 = 30%) (P-value = 0.013, Fisher’s exact test). Remarkably, among the 135 PNTmEs that we filtered out, only 10 (or 7.4%) were true essential, significantly lower than that of the original TmEs, i.e., 30% (P-value <1E-8). On the other hand, among the 12 PETmNs, 5 (or 42%) were true essential, significantly higher than that in the original TmNs (2.3%) (P-value <1E-6). These results strongly indicated that our model successfully enhanced the accuracy of the original TM assignments.

**Table 2:** Improvement of overlaps with the PEC dataset using our model.

TM dataset	Our Statistical Model	PEC dataset (Gold Standard)	
(Gerdes set)		E (259)	N (3574)
TmEs (615)	PETmEs (480)	176	304
	PNTmEs (135)	10	125
TmNs (3218)	PETmNs (12)	5	7
	PNTmNs (3206)	68	3138

**Figure 3:**
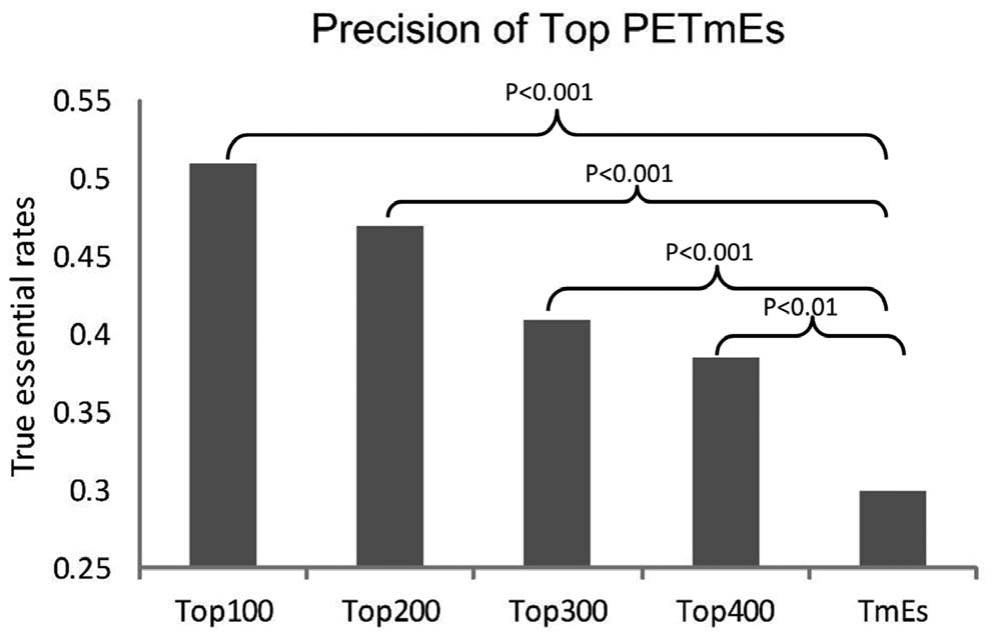
Enrichment of true essential genes using different thresholds of the confidence score.

Our results also showed a positive correlation between the confidence score and the enrichment of essential genes (**Fig. 3**). In other words, the higher confidence scores we chose as the cutoff, the higher percentage of true essential genes our predictions contained, indicating that our score system is in agreement with the distribution of essential genes. For instance, the top 100 PETmEs contained 50 true essential genes, this ratio is significantly higher than that in the original TmEs (30%) with P-value <1E-4.

### 5. Testing the Model’s Robustness in Analyzing Subsaturation Level TM Datasets

Compared with a saturated TM experiment, an unsaturated TM experiment generally contains a higher TmER, because genes are more likely to be missed by transposon insertions and thus incorrectly assigned as essential.

To test our model’s applicability to unsaturated TM datasets, we randomly removed 10%, 30% and 50% of the total insertions from the Gerdes dataset to simulate the effects of different subsaturation levels of TM experiments. We then applied our model on these subsaturated datasets.

**Figure 4:**
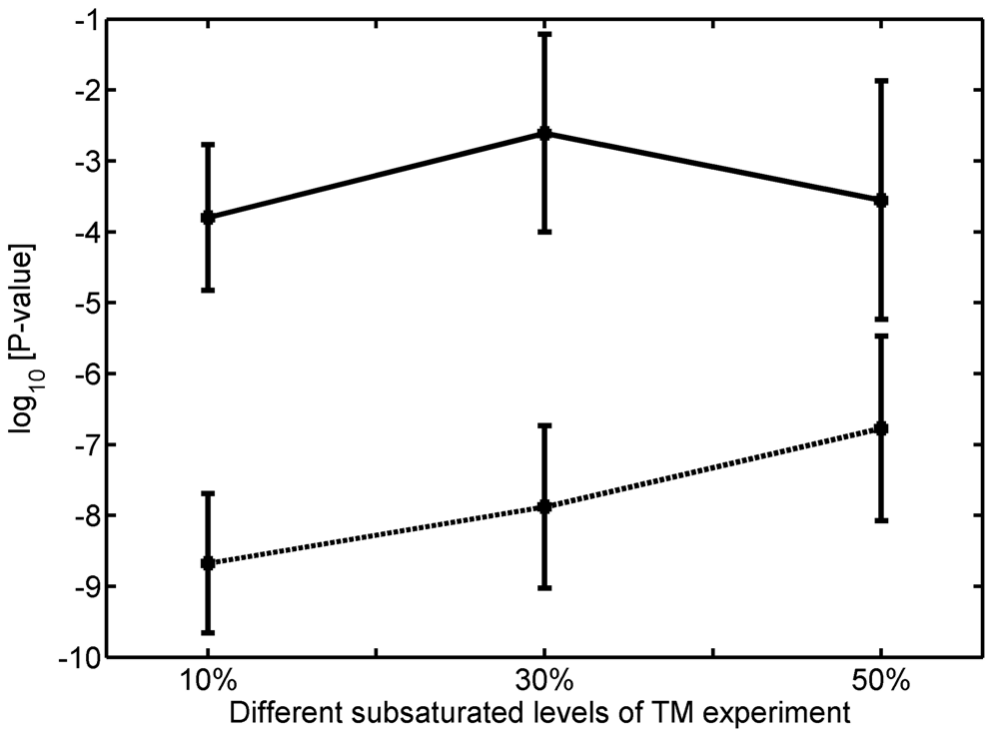
Robustness of our model at subsaturation levels of transposon insertions. The dashed line showed p-values of the Fisher’s exact test to examine whether the true essential rate in PNTmEs is significantly lower than that in the original TmEs set. Similarly, the solid line showed p-values of the Fisher’s exact test to examine whether the true essential rate in our PETmNs is significantly higher than that in the original TmNs set.

The results suggested a strong robustness in analyzing subsaturation level TM datasets (**Fig. 3**). As shown in **Fig. 4**, the lower curve (dashed line) showed the p-values of the Fisher’s exact test to examine whether the true essential rate in PNTmEs is significantly lower than that in the original TmEs set. Similarly, the upper curve (solid line) showed the p-values of the Fisher’s exact test to examine whether the true essential rate in our PETmNs is significantly higher than that in the original TmNs set. For each of the three (10%, 30% and 50%) random experiments, we repeated 100 times to obtain the error bars. The results showed that under each of these subsaturation conditions, our model still significantly improved TM results.

### 6. Testing the Model’s Applicability to *P. aeruginosa* by Allelic Exchange Experiments

The ultimate test is to see if our model is applicable to other microorganisms. A set of essential genes has been determined by TM to a saturated level in another γ-Proteobacteria, *P. aeruginosa PAO1* (Jacobs dataset) [29]. This TM dataset contains 678 putative essential genes and 4892 non-essential genes. If we assume the probability that an individual transposon insertion disrupts a gene’s function, is the same across different species, i.e., *P*_1_ = 0.942 and *P*_2_ = 0.984 as those in *E. coli*, then the overall essential rate 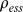 we estimated for *PAO1* is 10.1% and the expected numbers of PETmEs and PETmNs are 540 and 15, respectively.

Because a whole-genome single gene knockout dataset is not yet available in this organism, we chose to pursue allelic exchange experiments to validate our predictions in *PAO1*.

In order to make sure our experimental procedure can correctly identify essential genes, we first tested it on PA4238 as positive control. PA4238 is a subunit of RNA polymerase, which is the target of the microbicidal antibiotic, Rifampin [39]. The essentiality of PA4238 is confirmed by independent gene knockout efforts [24], [29]. As expected, PA4238 was determined by the allelic exchange experiments as essential. According to our model, PA4238 has a rank 46 out of 678, higher than the expected number of essential genes among TmEs, i.e., 540; therefore it was predicted as essential by our model. In this case, the results from the TM assignment, our model and the allelic exchange experiment are consistent (**Table 3**).

**Table 3:** Validation using allelic exchange experiments in *Pseudomonas aeruginosa PAO1*. E – Essential; N – Non-essential.

*PAO1* genes	Length	Local Insertion Density	Assignments by TM	Ranks by Our Model	Assignments by our model	Assignments by Allelic exchange experiments
PA3746	1374	4.9521	N	8/4289	E	E
PA4260	822	3.4102	N	103/4289	N	E
PA0985	1497	7.5415	N	113/4289	N	N
PA4238	1002	3.3564	E	46/678	E	E (Positive Control)
PA0723	249	7.4446	E	414/678	E	E
PA2954	570	2.0336	E	588/678	N	N
PA2143	288	2.1479	E	663/678	N	N

We then selected six ORFs from the *PAO1* TM dataset to further test our model: PA0723, PA2954 and PA2143 were selected from the TmEs; and PA3746, PA4260 and PA0985 were selected from the TmNs. The main purpose to conduct allelic exchange experiments for validations was to demonstrate that TM indeed contains errors and our method is capable of correcting some of these errors. To serve this purpose, we chose the genes that are most likely to be TM errors as our testing cases based on the following considerations: First, the testing cases among TmEs should be lower ranked (close to or below the cutoff of 540) while the testing cases among TmNs should be higher ranked (close to or above the cutoff 15). The higher-ranked genes among TmEs and lower-ranked genes among TmNs are mostly agreements between TM and our model and most likely to be correct assignments; therefore, they would not be interesting testing cases. Second, among these lower-ranked TmEs and higher-ranked TmNs, we challenged ourselves by selecting those more difficult cases, i.e., we either cannot find an ortholog in *E. coli* or the assignments based on orthologs are contradictory to TM assignments. Third, we chose our testing cases to be on both sides of the cutoffs to test the robustness of our cutoff setting. Finally, among the short list of genes that met the above three criteria, we nailed down to six based on experimental feasibility.

Among the three TmEs (PA2954, PA0723 and PA2143), PA0723 was a true essential gene but PA2954 and PA2143 turned out to be non-essential. According to our model, PA0723 was ranked 414 out of 678. Because the expected number of essential genes among TmEs is 540, it was correctly predicted to be essential. In contrast, PA2954 and PA2143 were given a rank of 588 and 663, respectively. They were both predicted as false positive error (i.e., non-essential) genes because their ranks were lower than 540. Our model was correct in all three cases.

Among the three TmNs (PA3746, PA4260 and PA0985), PA0985 was a true non-essential gene but PA3746 and PA4260 were tested to be essential. Our model assigned PA3746 a rank of 8 out of 4892. Because the rank was higher than 15, the expected number of essential genes among the TmNs, PA3746 was predicted to be essential. In contrast, PA0985 and PA4260 were assigned a rank of 113 and 103, respectively; they were predicted to be non-essential. Our model was correct in two out of the three cases.

Overall, among the seven genes tested by allelic exchange experiments, our model agreed with the experimental validations in six of them. Remarkably, among all three cases that directly contradicted the TM assignments, allelic exchange experiments supported our predictions. Details of allelic exchange experiments are provided in the **Supporting Information** (**Fig. S2** and **Table S3**).

### 7. Examples of Identified TM Annotation Errors Confirmed by Literature

Among the TM annotation errors in *PAO1* identified by our model, a number of them have been confirmed by literature. Below are three examples:

Hfq (PA4944) is a RNA-binding protein in *PAO1* and involved in the Bacterial RNA degradation pathway. It is identified as an essential gene by TM experiments [29], but assigned as non-essential by our model (ranked 572 out of the 678 TmEs). A recent study [40] investigated the effect of Hfq on virulence and stress response of *PAO1* and compared the growth rate of a wild-type strain and a Δhfq strain. The Δhfq strain showed a reduced growth rate compared with the wild-type strain, which indicates that this gene is essential for fitness but not for survival and should be referred as a non-essential gene here.

FpvI (PA2387) is a RNA polymerase sigma factor and also a TM-assigned essential gene in *PAO1*. Our model predicted it as non-essential by assigning it a rank of 582 out of the 678 TmEs. Beare et.al studied its role in siderophore-mediated cell signaling [41]. They found that FpvI is required for the synthesis of ferripyoverdine receptor FpvA which is part of the signaling pathway regulating pyoverdine (a form of siderophore) production. In the experiment, they constructed a ΔfpvI strain and found that this mutant produced much lower amounts of FpvA than the wild-type stain, which suggests that FpvI is required for normal amounts of FpvA production but not for survival.

PcrG (PA1705) is a regulator in type III secretion system (TTSS) which enables the bacteria to translocate virulence effectors directly into the cytosol of host cells. It ranks 559 out of the 678 TmEs by our model and is predicted as a non-essential gene, contradicting the TM assignment. To further understand its mechanism, Sundin’s group constructed an in-frame deletion mutant of PcrG and cultured the mutant in LB medium. They found the PcrG mutant can up-regulate the expression of exoenzyme S (ExoS) which has been identified as effectors targeted into host cells by the TTSS of *PAO1* [42]. This experiment also proved that PcrG is a non-essential gene.

### 8. Testing the Model’s Broad Applicability to Other Organisms

*Francisella tularensis* is a Gram-negative, intracellular pathogen that causes the disease tularemia. *F. tularensis novicida* is commonly used as surrogate in virulence studies particularly virulent toward mice and is also able to cause tularemia-like disease in rodents. Genome-scale TM experiments have been performed on its subspecies *F. tularensis novicida* [25]. This TM library consists of 16,508 unique insertions covering 1,434 out of the 1,767 genes, an average of >9 insertions per gene, which achieves the highest coverage among the available TM experiments of bacterial species. We applied our model on this TM library and estimated the overall essential rate is 14.5%. Among the 333 TmEs, we predicted 251 genes as essential labeled them as PETmEs. Among the 1434 TmNs, we predicted 5 as essential and labeled as PETmNs. The prediction results have been made available on the Web server described in the next section. The TM annotations from other microorganisms will be processed and deposited in our Web server once the results become available.

### 9. Developing a Web Server that Provides Convenient Access to Our Method

**Figure 5:**
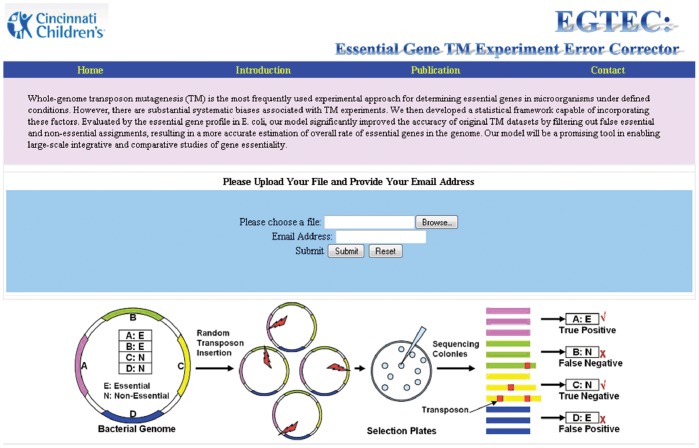
Interface of the EGTEC Web server.

To make our model readily available to the research community, we developed a convenient and user-friendly Web service named EGTEC (The Essential Gene TM Annotation Error Corrector) hosted at: http://research.cchmc.org/essentialgene/. The EGTEC interface is implemented using PHP-HTML and comprises of two main functions (**Fig. 5**):

1. A query tool for a quick exploration of the corrected TM sample results in bacteria, which currently includes *E. coli*, *P. aeruginosa* and *F. tularensis novicida*. Once a query is received, the tool will check for the gene’s presence in the database and displays the matching records for the input, including the original TM annotations and the EGTEC’s prediction as well as the results from single-gene knockout experiments if available. The tool will accept the input query gene’s name in the following formats: gene symbol/name, GenBank accession number or Entrez Gene ID.
2. An uploading tool for submitting new TM experimental data by users. The EGTEC accepts user-generated TM experimental results and makes corrections on these results. The input should contain the following information: (a) Each ORF’s name, (b) Start and end position of this ORF and (c) The transposon insert position in this ORF. A sample input file is provided along with the uploading box. When finished, the corrected TM annotations including the predicted probability of each ORF being essential will be sent to the user through email.

## Discussion

The intrinsic biases in TM experiments motivated us to develop a statistical framework to systematically filter out errors (both FETmEs and FNTmNs) and thus improved the accuracy in TM-determined essential gene annotations. This model is significant in four ways:

First, our model significantly enhances the accuracy of the original TM assignments. In the *E. coli* TmEs, our PNTmEs, i.e., the false essential genes filtered out by our method, had a significantly lower true essential rate than that in the original TmEs. In contrast, in the TmNs, our PETmNs, i.e., the predicted essential genes from TmNs, had a significantly higher true essential rate than that in the original TmNs. The confidence scores generated by our model were shown to have positive correlations with the enrichment of true essential genes.

Being able to assign PETmNs demonstrates the advantage of our approach over previous studies by recovering true essential genes from TmNs. Most of the existing models, e.g., in [28], only focused on removing false essential genes from TmEs, but are incapable of recovering false non-essential genes, although relatively few, from TmNs. In *E. coli*, among the 12 false non-essential genes we recovered, five are true essential, significantly higher than original TmNs (**Table 2**).

Second, because our model adopts simple but more realistic assumptions, it is applicable across multiple microorganisms. It is very important to note that our method is not dependent on single gene knockout results to make predictions. The single gene knockout results in *E. coli* were only used for assessing the TM errors and evaluating the performance of our predictions. In *P. aeruginosa PAO1* where single gene knockout results are unavailable, we demonstrated that our method is remarkably accurate based on literature and the six allelic exchange experiments. Among the seven chosen genes for an experimental test, six of them were assigned correctly by our model with an overall accuracy of 86%. These results clearly demonstrated our method’s reliability and applicability to organisms that do not have single gene knockout results. We further demonstrated our model’s broad applicability to *F. tularensis novicida*, where single gene knockout results are also unavailable. Therefore, we believe, if granted full access to the information of transposon insertion positions in the bacterial genomes, our model can be readily applicable to all 24 available TM datasets listed in **Table S1**.

Third, our model displays robustness in analyzing unsaturation level TM datasets and resisting with experimental errors as demonstrated in the simulated unsaturation level TM datasets. This is potentially useful in significantly reducing the time and costs currently associated with TM experiments.

Finally, our model is flexible and able to incorporate more potential factors. For example, we assigned different weights to the insertions based on the positions where they were inserted in the genes. In the future, we will consider other factors that might affect the accuracy of TM experiments [33].

To benchmark our method’s performance against different types of computational methods, we compared their prediction performance in **Table S4**. We only included the studies conducted in *E. coli* in order to make an objective comparison. It is not surprising that supervised methods that rely on gold standard datasets often outperform non-supervised methods that do not rely on gold standard datasets. The main problem of supervised learning is that it is not applicable to understudied species where very few essential genes are already known; therefore, its superior performance in sensitivity, specificity and precision is not meaningful to the problem we intend to solve. Although the constraint-based method outperforms our model in precision, its sensitivity and specificity are rather poor. This reflects its limitation of being dependent on *a priori* knowledge on pathways and reactions. Homology mapping has a similar level of performance as our model, but it requires a closely related model organism, and the accuracy of assignment is highly dependent on the distance between the model organism and the target organism. In this case, homology mapping also performs well mainly because single gene knockout results are available in a well-studied and closely-related organism, *Acinetobacter baylyi* [43]. It is unrealistic to expect such a model organism is always available for an understudied species.

Our approach represents a significant advancement over Gerdes approach to filter TM errors. First, the cutoff settings in Gerdes approach appeared to be somewhat arbitrary and lack of statistical rigorousness. For example, all genes longer than 240 bp and free from inserts were assigned as essential, and genes with at least one insertion were designated as non-essential unless they are relatively long (>900 bp). In contrast, all of our parameters were determined through the iterative learning process. Second, Gerdes annotations were not strictly based on TM data but also involved manual inspection that requires prior knowledge on *E. coli* physiology. Therefore, Gerdes approach cannot be easily extended to an understudied organism and expected to achieve the same performance. In contrast, our approach only relies on the TM data, i.e., transposon insertion positions in the bacterial genome. Even without using prior knowledge to improve our assignments, our model still significantly outperformed Gerdes approach in precision and specificity (**Table S4**).

In other organisms, although the performance metrics cannot be easily assessed because definite mappings of essential genes are not yet available in those species, our approach exhibits several advantages: Our model is capable of estimating the probability score of being essential for individual genes, rather than only estimating an overall essential rate for the whole genome as in Jacobs *et al.*’s method. In addition, Jacobs *et al.* used a multinomial distribution which cannot incorporate the difference between “hot” and “cold” spots. Furthermore, their method is not applicable for correcting false negative errors, i.e., detecting true essential genes among TmNs. In Lamichhane *et al.*’s study, their method was applicable to a subsaturation level of TM. Since a gold-standard dataset is not yet available in *M. tuberculosis,* we cannot compare our subsaturation results with theirs. In addition, they also have the same issues as in Jacobs *et al.*’s method by disregarding false negative errors and differences in insertion density. By taking into account these features, our model is expected to be more realistic and accurate.

Our effort is timely given the large number of existing annotations of essential genes and rapidly increased TM datasets. The need for such a system to assess the TM errors becomes even more urgent as researchers start to explore conditional essentials, which could hold the key to understanding gene essentiality. For the past three years, we have seen numerous new bacterial essential gene sets published and the majority of them were generated by TM approach [44], [45], [46], [47], [48], [49], [50], [51], [52], [53] (**Table S1**). We expect growth of essential gene datasets to greatly accelerate. Therefore our contribution is not only valuable but also timely. As long as TM experiments remain the dominant method to determine essential gens in prokaryotes, our method is expected to remain useful.

A logical next step is to investigate our model’s applicability to eukaryotes. However, because eukaryotes have much more complex genome structure than prokaryotes, e.g., introns, the mechanism for eukaryotic essential genes is expected to be quite different from the prokaryotic essential genes. For the same reason, transposons may also likely work differently in eukaryotes than in prokaryotes. A thorough investigation on these aspects should be performed before we apply our model to eukaryotic genomes.

In summary, we have developed a promising tool that is crucial for large-scale data mining on essential genes. The analysis enabled by this tool will eventually lead to a better understanding on the mechanistic basis underlying gene essentiality.

## Materials and Methods

### 1. Data Sources

In this study, we tested our model on three different prokaryotic organisms: *E. coli*, *P. aeruginosa PAO1* and *F. tularensis novicida*. We chose to study these three organisms because their genomic sequences and the TM datasets are publicly available.

The genomic and protein sequences of *E. coli* MG1655 were downloaded from the Comprehensive Microbial Resource (CMR) at http://cmr.jcvi.org/. It contained 4,289 protein coding genes in total.

The *E. coli* essential dataset was downloaded from Profiling of *E. coli* Chromosome (PEC) v4 at http://www.shigen.nig.ac.jp/. This dataset (PEC set) contained 302 essential genes and 4477 non-essential genes.

The *E. coli* TM dataset was downloaded from [23]. Of the total 4291 protein-coding genes in this dataset, 3,311 ORFs have observed transposon insertions and 649 ORFs have not. The remaining 331 genes were excluded from analysis because no reliable PCR data can be obtained for the corresponding region of the *E. coli* chromosome for the technical reasons.

The total 5,568 protein sequences of *P. aeruginosa PAO1* were downloaded from http://www.pseudomonas.com/(Pseudomonas_aeruginosa_PAO1.faa, revision 2009-07-17).

The *P. aeruginosa PAO1* TM dataset was downloaded from [29]. This dataset contained 4,892 ORFs that have observed transposon insertions and 678 ORFs that have not.

The *F. tularensis novicida* TM dataset was downloaded from [25]. This datasets contain 333 putative essential genes and 1,767 putative non-essential genes.

The *E. coli* operon dataset was downloaded from Regulon DB version 6.4 at http://regulondb.ccg.unam.mx/. This dataset contained 2,665 operons that inferred experimentally or computationally in the *E. coli* genome.

### 2. Simulation of Random Insertions

Assuming a uniform distribution for the transposon insertions within an ORF, we conducted a simulation experiment for random insertions. In the Gerdes set, we observed 12,966 total transposon insertions inside ORFs, excluding intergenic insertions. We randomly generated 12,966 insertions along the genome, excluding intergenic regions. For each random insertion, we recorded its relative position inside the ORF. Repeating this simulation 1,000 times, we obtained the distribution of the positions of simulated insertions. We then evaluated the empirical insertion distribution of TmNs (both TNTmNs and FNTmNs) by calculating the probability *P* for the randomized insertions appearing in a certain position for an equal or greater number of times than in the real experiment. We concluded that there was a significant difference if the P-values ≤0.01 (**Table S2**).

### 3. Derivation of Equations

Based on the probability theory, Eq. (1) can be rewritten into two parts (**Fig. 2**):
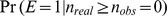


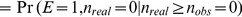
(PartA)

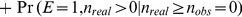
(PartB)


Part A:
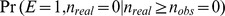


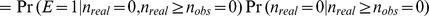
where 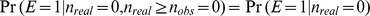 is the probability that the gene is essential given the actual insertion number equal to 0. This means it never had any insertion and was missed by all transposons by chance. Thus, we cannot further infer the information about this gene, we assume its probability of being essential should equal to the overall percentage of essential genes in the genome. We thus have:
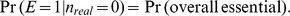



Since the transposon insertion process follows a Poisson distribution, we get:
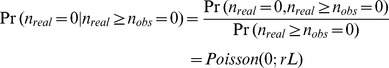



Part B:
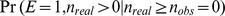


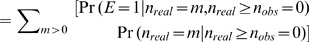



where 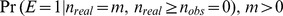 is the probability that this gene is essential given that the real insertion number is *m* (*m* >0), and none of these insertions survived. Since we didn’t observe any insertion, all *m* insertions should be effective; otherwise, mutants with ineffective insertions should have survived. In this case, the target gene is always essential, as all insertions interrupted its function and caused the mutant’s death. Thus, we have:
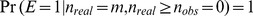


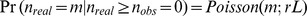



Similarly, Eq. (2) can also be rewritten into two parts (**Fig. 2**):
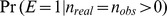


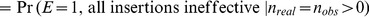
(PartC)

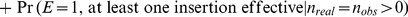
(PartD)


Part C:

Since all the insertions have not disrupted this gene’s function and we cannot further infer the information about this gene, we assume its probability of being essential should equal to the overall percentage of essential genes in the genome, thus,
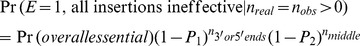



Part D:

Since we observed effective insertions in the target gene and the mutant with this gene disrupted still survived, the target gene cannot be essential.
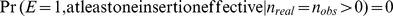



### 4. Estimating Parameters Using an Iterative Procedure

We iteratively estimated the unknown parameters 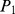 (the probability that an individual insertion disrupts a gene’s function when it occurs within the 25% extreme ends of a gene), 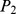 (the probability that an individual insertion disrupts a gene’s function when it occurs in the middle of a gene) and 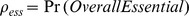 (the true essential rate in the genome) as follows:

Step 1. Based on the model assumptions, we defined the empirical estimators for 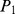 and 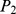 by calculating the number of corresponding insertions (denominator) and their effectiveness (numerator) using the definition as follows:
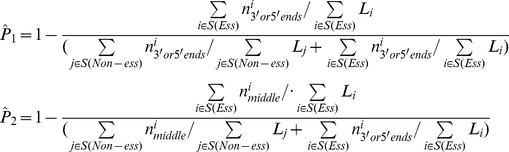
(3)


Here 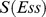 and 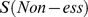 denote the set of essential and non-essential genes at the current step, respectively. 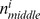 is the observed insert number within the middle regions of the *i* ^th^ gene; 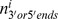 is the observed insert number at the 3′ or 5′ends and *L_i_* is the length of that gene.

Step 2. Using 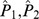 to estimate 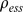:

In this step, first, we calculated the expected number of essential genes in TmEs and TmNs respectively, by the following equation:
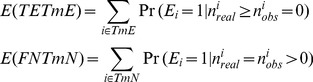
(4)


Then 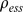 can be estimated by:
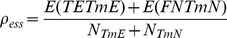
(5)


Substituting (4) into (5), we can get:
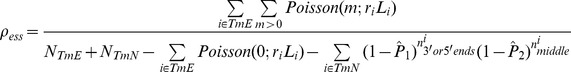
(6)


Step 3. Using the current 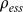 and 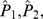 to assign an essential probability score to each individual gene in the TM dataset.

Step 4. Ranking these essential probability scores in TmEs set and using the expected number of essential genes in that dataset as the cutoff, the top genes are considered as PETmEs. Similarly, ranking the essential probability scores in TmNs dataset and using the expected number of essentials in that dataset as the cutoff, the top genes are considered as the PETmNs, so our filtered essential dataset should be the combination of the PETmEs and PETmNs.

Step 5. Updating the current essential dataset 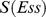 using this filtered essential dataset and the remaining genes to update the current non-essential dataset 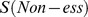.

Step 6. Going back to the Step 1 until the result converges.

In our model, the initial *P_1_* and *P_2_* were randomly assigned between 0 and 1, and updated during the iteration process until converge.

### 5. Experimental Validation in *P. aeruginosa PAO1*

To verify our predictions, we conducted allelic exchange experiments on the seven chosen genes: PA4238, PA0723, PA0985, PA2954, PA2143, PA3746 and PA4260. First, using genomic DNA from strain *PAO1* as template and standard PCR techniques, we cloned the PCR fragments containing the desired genes and 1 kb of flanking DNA into pUC19. We next inserted a non-polar *aac*C1 (Gm^R^) cassette from pUCGM [54] into the target gene in the same transcriptional orientation to ensure that there was no polar effects on the downstream genes. The inserted fragments were then subcloned into the gene replacement vector, pEX100T, and the resultant constructs were conjugated into *PAO1* with selection for Gm^R^. Transconjugates were resolved on LB agar containing 7% sucrose as a counter-selectable marker. Isogenic mutants were confirmed initially by PCR and then Southern blot. Complementation was both via single copy using the unique *attB* locus as well as via stable plasmids (details in **Text S1**).

## Supporting Information

Figure S1 **Identification of essential genes by TM.**
(DOC)

Figure S2 **PCR analysis of PA0985 merodiploid (single crossover) or mutant (double crossover) recombinant strains.**
(DOCX)

Table S1 (DOC)

Table S2 (DOC)

Table S3 (DOC)

Table S4 (DOC)

Text S1 (DOC)

## References

[1] Dowell RD, Ryan O, Jansen A, Cheung D, Agarwala S, et al (2010) Genotype to phenotype: a complex problem. Science 328: 469. 20413493 10.1126/science.1189015 PMC4412269

[2] Kato J, Hashimoto M (2007) Construction of consecutive deletions of the Escherichia coli chromosome. Mol Syst Biol 3: 132. 17700540 10.1038/msb4100174 PMC1964801

[3] Winzeler EA, Shoemaker DD, Astromoff A, Liang H, Anderson K, et al (1999) Functional characterization of the S. cerevisiae genome by gene deletion and parallel analysis. Science 285: 901–906. 10436161 10.1126/science.285.5429.901

[4] Mushegian A (1999) The minimal genome concept. Curr Opin Genet Dev 9: 709–714. 10607608 10.1016/s0959-437x(99)00023-4

[5] Arigoni F, Talabot F, Peitsch M, Edgerton MD, Meldrum E, et al (1998) A genome-based approach for the identification of essential bacterial genes. Nat Biotechnol 16: 851–856. 9743119 10.1038/nbt0998-851

[6] Bruccoleri RE, Dougherty TJ, Davison DB (1998) Concordance analysis of microbial genomes. Nucleic Acids Res 26: 4482–4486. 9742253 10.1093/nar/26.19.4482 PMC147859

[7] Joyce AR, Palsson BO (2008) Predicting gene essentiality using genome-scale in silico models. Methods Mol Biol 416: 433–457. 18392986 10.1007/978-1-59745-321-9_30

[8] Perumal D, Samal A, Sakharkar KR, Sakharkar MK (2011) Targeting multiple targets in Pseudomonas aeruginosa PAO1 using flux balance analysis of a reconstructed genome-scale metabolic network. J Drug Target 19: 1–13. 20233082 10.3109/10611861003649753

[9] Plata G, Hsiao TL, Olszewski KL, Llinas M, Vitkup D (2010) Reconstruction and flux-balance analysis of the Plasmodium falciparum metabolic network. Mol Syst Biol 6: 408. 20823846 10.1038/msb.2010.60 PMC2964117

[10] Imam S, Yilmaz S, Sohmen U, Gorzalski AS, Reed JL, et al (2011) iRsp1095: a genome-scale reconstruction of the Rhodobacter sphaeroides metabolic network. BMC Syst Biol 5: 116. 21777427 10.1186/1752-0509-5-116 PMC3152904

[11] Chen Y, Xu D (2005) Understanding protein dispensability through machine-learning analysis of high-throughput data. Bioinformatics 21: 575–581. 15479713 10.1093/bioinformatics/bti058

[12] Saha S, Heber S (2006) In silico prediction of yeast deletion phenotypes. Genet Mol Res 5: 224–232. 16755513

[13] Gustafson AM, Snitkin ES, Parker SC, DeLisi C, Kasif S (2006) Towards the identification of essential genes using targeted genome sequencing and comparative analysis. BMC Genomics 7: 265. 17052348 10.1186/1471-2164-7-265 PMC1624830

[14] Seringhaus M, Paccanaro A, Borneman A, Snyder M, Gerstein M (2006) Predicting essential genes in fungal genomes. Genome Res 16: 1126–1135. 16899653 10.1101/gr.5144106 PMC1557763

[15] Deng J, Tan L, Lin X, Lu Y, Lu LJ (2012) Exploring the optimal strategy to predict essential genes in microbes. Biomolecules: 1–22. 10.3390/biom2010001 PMC4030871 24970124

[16] Deng J, Deng L, Su S, Zhang M, Lin X, et al (2011) Investigating the predictability of essential genes across distantly related organisms using an integrative approach. Nucleic Acids Res 39: 795–807. 20870748 10.1093/nar/gkq784 PMC3035443

[17] Judson N, Mekalanos JJ (2000) Transposon-based approaches to identify essential bacterial genes. Trends Microbiol 8: 521–526. 11121763 10.1016/s0966-842x(00)01865-5

[18] Winsor GL, Lam DK, Fleming L, Lo R, Whiteside MD, et al (2011) Pseudomonas Genome Database: improved comparative analysis and population genomics capability for Pseudomonas genomes. Nucleic Acids Res 39: D596–600. 20929876 10.1093/nar/gkq869 PMC3013766

[19] Zhang R, Lin Y (2009) DEG 5.0, a database of essential genes in both prokaryotes and eukaryotes. Nucleic Acids Res 37: D455–458. 18974178 10.1093/nar/gkn858 PMC2686491

[20] Chen WH, Minguez P, Lercher MJ, Bork P (2012) OGEE: an online gene essentiality database. Nucleic Acids Res 40: D901–906. 22075992 10.1093/nar/gkr986 PMC3245054

[21] Berg DE, Howe MM (1989) Mobile DNA. Washington, D.C.: American Society for Microbiology. xii, 972 p., [975] p. of plates p.

[22] Hamer L, DeZwaan TM, Montenegro-Chamorro MV, Frank SA, Hamer JE (2001) Recent advances in large-scale transposon mutagenesis. Curr Opin Chem Biol 5: 67–73. 11166651 10.1016/s1367-5931(00)00162-9

[23] Gerdes SY, Scholle MD, Campbell JW, Balazsi G, Ravasz E, et al (2003) Experimental determination and system level analysis of essential genes in Escherichia coli MG1655. J Bacteriol 185: 5673–5684. 13129938 10.1128/JB.185.19.5673-5684.2003 PMC193955

[24] Liberati NT, Urbach JM, Miyata S, Lee DG, Drenkard E, et al (2006) An ordered, nonredundant library of Pseudomonas aeruginosa strain PA14 transposon insertion mutants. Proc Natl Acad Sci U S A 103: 2833–2838. 16477005 10.1073/pnas.0511100103 PMC1413827

[25] Gallagher LA, Ramage E, Jacobs MA, Kaul R, Brittnacher M, et al (2007) A comprehensive transposon mutant library of Francisella novicida, a bioweapon surrogate. Proc Natl Acad Sci U S A 104: 1009–1014. 17215359 10.1073/pnas.0606713104 PMC1783355

[26] Akerley BJ, Rubin EJ, Novick VL, Amaya K, Judson N, et al (2002) A genome-scale analysis for identification of genes required for growth or survival of Haemophilus influenzae. Proc Natl Acad Sci U S A 99: 966–971. 11805338 10.1073/pnas.012602299 PMC117414

[27] Glass JI, Assad-Garcia N, Alperovich N, Yooseph S, Lewis MR, et al (2006) Essential genes of a minimal bacterium. Proc Natl Acad Sci U S A 103: 425–430. 16407165 10.1073/pnas.0510013103 PMC1324956

[28] Lamichhane G, Zignol M, Blades NJ, Geiman DE, Dougherty A, et al (2003) A postgenomic method for predicting essential genes at subsaturation levels of mutagenesis: application to Mycobacterium tuberculosis. Proc Natl Acad Sci U S A 100: 7213–7218. 12775759 10.1073/pnas.1231432100 PMC165855

[29] Jacobs MA, Alwood A, Thaipisuttikul I, Spencer D, Haugen E, et al (2003) Comprehensive transposon mutant library of Pseudomonas aeruginosa. Proc Natl Acad Sci U S A 100: 14339–14344. 14617778 10.1073/pnas.2036282100 PMC283593

[30] Hutchison CA, Peterson SN, Gill SR, Cline RT, White O, et al (1999) Global transposon mutagenesis and a minimal Mycoplasma genome. Science 286: 2165–2169. 10591650 10.1126/science.286.5447.2165

[31] Blades NJ, Broman KW (2002) Estimating the number of essential genes in a genome by random transposon mutagenesis. Technical Report MS02–20, Department of Biostatistics, Johns Hopkins University Working Paper 15.

[32] Gerdes S, Edwards R, Kubal M, Fonstein M, Stevens R, et al (2006) Essential genes on metabolic maps. Curr Opin Biotechnol 17: 448–456. 16978855 10.1016/j.copbio.2006.08.006

[33] Gerdes SY, Scholle MD, D’Souza M, Bernal A, Baev MV, et al (2002) From genetic footprinting to antimicrobial drug targets: examples in cofactor biosynthetic pathways. J Bacteriol 184: 4555–4572. 12142426 10.1128/JB.184.16.4555-4572.2002 PMC135229

[34] Good IJ (1986) Some statistical applications of Poisson’s work. Statistical science 1 (2): 157–180.

[35] Ross SM (1996) Stochastic processes. New York: Wiley. xv, 510 p. p.

[36] Lehmann EL, Casella G (1998) Theory of point estimation. New York: Springer. xxvi, 589 p.p.

[37] Zolman JF (1993) Biostatistics : experimental design and statistical inference. New York: Oxford University Press. xv, 343 p.p.

[38] Balakrishnan N, Melas VB, Ermakov SM (2000) Advances in stochastic simulation methods. Boston: Birkhäuser. xxvi, 386 p.p.

[39] Knox C, Law V, Jewison T, Liu P, Ly S, et al (2011) DrugBank 3.0: a comprehensive resource for ‘omics’ research on drugs. Nucleic Acids Res 39: D1035–1041. 21059682 10.1093/nar/gkq1126 PMC3013709

[40] Sonnleitner E, Hagens S, Rosenau F, Wilhelm S, Habel A, et al (2003) Reduced virulence of a hfq mutant of Pseudomonas aeruginosa O1. Microb Pathog 35: 217–228. 14521880 10.1016/s0882-4010(03)00149-9

[41] Beare PA, For RJ, Martin LW, Lamont IL (2003) Siderophore-mediated cell signalling in Pseudomonas aeruginosa: divergent pathways regulate virulence factor production and siderophore receptor synthesis. Mol Microbiol 47: 195–207. 12492864 10.1046/j.1365-2958.2003.03288.x

[42] Sundin C, Thelaus J, Broms JE, Forsberg A (2004) Polarisation of type III translocation by Pseudomonas aeruginosa requires PcrG, PcrV and PopN. Microb Pathog 37: 313–322. 15619427 10.1016/j.micpath.2004.10.005

[43] de Berardinis V, Vallenet D, Castelli V, Besnard M, Pinet A, et al (2008) A complete collection of single-gene deletion mutants of Acinetobacter baylyi ADP1. Mol Syst Biol 4: 174. 18319726 10.1038/msb.2008.10 PMC2290942

[44] Chaudhuri RR, Allen AG, Owen PJ, Shalom G, Stone K, et al (2009) Comprehensive identification of essential Staphylococcus aureus genes using Transposon-Mediated Differential Hybridisation (TMDH). BMC Genomics 10: 291. 19570206 10.1186/1471-2164-10-291 PMC2721850

[45] Christen B, Abeliuk E, Collier JM, Kalogeraki VS, Passarelli B, et al (2011) The essential genome of a bacterium. Mol Syst Biol 7: 528. 21878915 10.1038/msb.2011.58 PMC3202797

[46] Lamichhane G, Freundlich JS, Ekins S, Wickramaratne N, Nolan ST, et al (2011) Essential metabolites of Mycobacterium tuberculosis and their mimics. MBio 2: e00301–00310. 21285434 10.1128/mBio.00301-10 PMC3031304

[47] Langridge GC, Phan MD, Turner DJ, Perkins TT, Parts L, et al (2009) Simultaneous assay of every Salmonella Typhi gene using one million transposon mutants. Genome Res 19: 2308–2316. 19826075 10.1101/gr.097097.109 PMC2792183

[48] Mendum TA, Newcombe J, Mannan AA, Kierzek AA, McFadden J (2011) Interrogation of global mutagenesis data with a genome scale model of Neisseria meningitidis to assess gene fitness in vitro and in sera. Genome Biol 12: R127. 22208880 10.1186/gb-2011-12-12-r127 PMC3334622

[49] Molina-Henares MA, de la Torre J, Garcia-Salamanca A, Molina-Henares AJ, Herrera MC, et al.. (2010) Identification of conditionally essential genes for growth of Pseudomonas putida KT2440 on minimal medium through the screening of a genome-wide mutant library. Environ Microbiol. 10.1111/j.1462-2920.2010.02166.x 20158506

[50] Murray GL, Morel V, Cerqueira GM, Croda J, Srikram A, et al (2009) Genome-wide transposon mutagenesis in pathogenic Leptospira species. Infect Immun 77: 810–816. 19047402 10.1128/IAI.01293-08 PMC2632054

[51] Soemphol W, Deeraksa A, Matsutani M, Yakushi T, Toyama H, et al (2011) Global analysis of the genes involved in the thermotolerance mechanism of thermotolerant Acetobacter tropicalis SKU1100. Biosci Biotechnol Biochem 75: 1921–1928. 21979075 10.1271/bbb.110310

[52] Stahl M, Stintzi A (2011) Identification of essential genes in C. jejuni genome highlights hyper-variable plasticity regions. Funct Integr Genomics 11: 241–257. 21344305 10.1007/s10142-011-0214-7

[53] Xu P, Ge X, Chen L, Wang X, Dou Y, et al (2011) Genome-wide essential gene identification in Streptococcus sanguinis. Scientific Reports 1: 125. 22355642 10.1038/srep00125 PMC3216606

[54] Schweizer HD (1993) Small broad-host-range gentamycin resistance gene cassettes for site-specific insertion and deletion mutagenesis. Biotechniques 15: 831–834. 8267974

