# Supplementary figures and images for: A Statistical Framework for Improving Genomic Annotations of Prokaryotic Essential Genes
Source: PLoS One. 2013 Mar 8;8(3):e58178. doi: 10.1371/journal.pone.0058178 (PMC3592911; doi:10.1371/journal.pone.0058178)

**Figure S1**. Identification of essential genes by TM.


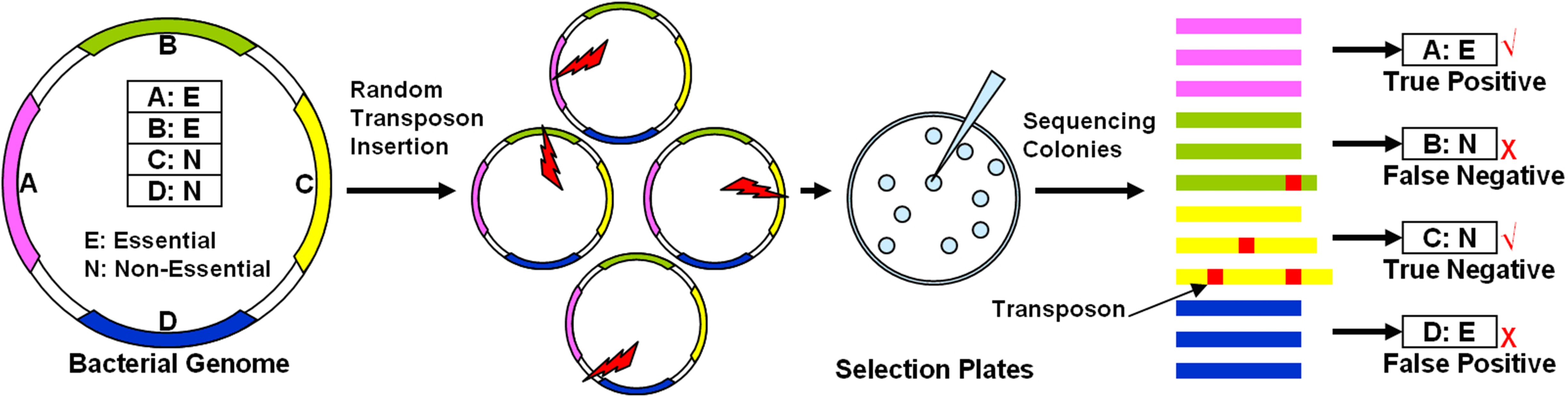

Supplement: Figure S1 — Identification of essential genes by TM. (DOC) [file pone.0058178.s001.doc]
