# Supplementary material for: A Statistical Framework for Improving Genomic Annotations of Prokaryotic Essential Genes
Source: PLoS One. 2013 Mar 8;8(3):e58178. doi: 10.1371/journal.pone.0058178 (PMC3592911; doi:10.1371/journal.pone.0058178)

**Figure S2**. PCR analysis of PA0985 merodiploid (single crossover) or mutant (double crossover) recombinant strains. WT, wild-type 1,497 bp PCR product of the PA0985 gene; 1 and 2, two merodiploid strains harboring wild-type and mutant copies; 3 and 4, mutant strains.


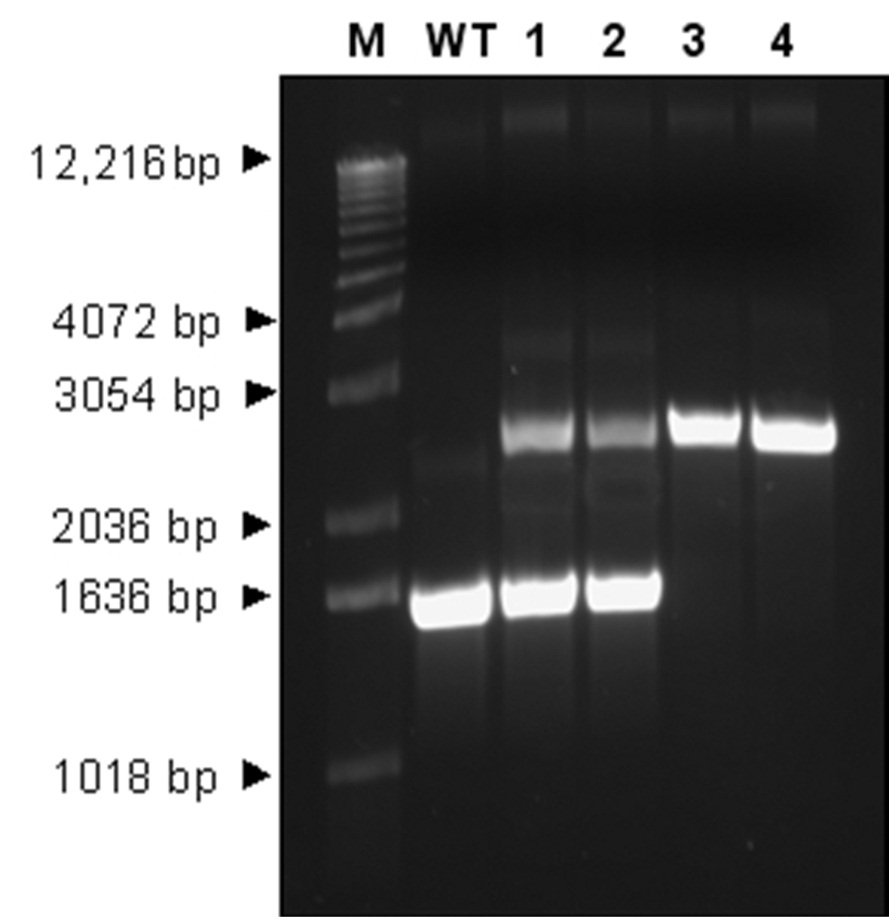

Supplement: Figure S2 — PCR analysis of PA0985 merodiploid (single crossover) or mutant (double crossover) recombinant strains. (DOCX) [file pone.0058178.s002.docx]
