# Supplementary material for: A Statistical Framework for Improving Genomic Annotations of Prokaryotic Essential Genes
Source: PLoS One. 2013 Mar 8;8(3):e58178. doi: 10.1371/journal.pone.0058178 (PMC3592911; doi:10.1371/journal.pone.0058178)
Supplement: Table S1 — (DOC) [file pone.0058178.s003.doc]

**Table S1.** Genomic scale TM studies in bacterial species.

| **Microorganism** | **Year** | **Number of Ess Genes** | **Growth Conditions** | **Ref.** |
| --- | --- | --- | --- | --- |
| *Mycoplasma genitalium* | 1999 | 265~ 350 | Laboratory growth conditions |  |
| *Escherichia coli* | 2001 | N/A | Luria-Bertani (LB) medium |  |
| *Haemophilus influenzae* | 2002 | 642 | Brain heart infusion (BHI) agar |  |
| *Escherichia coli* | 2003 | 620 | Luria-Bertani (LB) medium |  |
| *Mycobacterium tuberculosis* | 2003 | 614 | 7H10 agar (Difco) containing AD |  |
| *Pseudomonas aeruginosa PAO1* | 2003 | 678 | Luria-Bertani (LB) medium |  |
| *Escherichia coli* | 2004 | 73 anaerobic,  118 aerobic | Rich medium under aerobic or anaerobic growth conditions |  |
| *Helicobacter pylori 26695* | 2004 | 344 | Horse blood agar (HB) plates |  |
| *Mycoplasma genitalium* | 2006 | 381 | Laboratory growth conditions |  |
| *Pseudomonas aeruginosa UCBPP-PA14* | 2006 | 335 | Luria-Bertani (LB) medium |  |
| *Corynebacterium glutamicum* | 2006 | 650 | Complex medium |  |
| *P. aeruginosa strain PAO1* | 2006 | 24 | NY agar, anaerobic growth |  |
| *Francisella novicida* | 2007 | 392 | Trypticase soy broth |  |
| *Mycoplasma pulmonis UAB CTIP* | 2008 | 310 | Mycoplasma agar (MA) |  |
| *Vibrio cholerae N16961* | 2008 | 779 | Luria-Bertani (LB) medium |  |
| *SalmonelLa Typhi* | 2009 | 353 | Luria-Bertani (LB) medium |  |
| *Leptospira interrogans* | 2009 | N/A | Liquid EMJH medium |  |
| *Staphylococcus aureus* | 2009 | 351 | SOC medium |  |
| *Pseudomonas putida KT2440* | 2010 | 47 | Minimal medium with glucose |  |
| *Mycobacterium tuberculosis* | 2011 | 401 | Middlebrook 7H9 medium |  |
| *Caulobacter Crescentus* | 2011 | 480 | Rich media |  |
| *Acetobacter tropicalis SKU1100* | 2011 | 24 | YPG medium  Growth at high temperature |  |
| *Neisseria meningitidis* | 2011 | 585 | Rich CAB medium |  |
| *Campylobacter jejuni* | 2011 | 195 | Rich Mueller-Hinton medium |  |
