# Supplementary material for: A Statistical Framework for Improving Genomic Annotations of Prokaryotic Essential Genes
Source: PLoS One. 2013 Mar 8;8(3):e58178. doi: 10.1371/journal.pone.0058178 (PMC3592911; doi:10.1371/journal.pone.0058178)
Supplement: Table S2 — (DOC) [file pone.0058178.s004.doc]

**Table S2.** Comparing the position distribution of insertions with the random simulations.

| **Position**  (%)  **P-values** | [0,5) | [5,10) | [10,20) | [20,30) | [30,40) | [40,50) | [50,60) | [60,70) | [70,80) | [80,90) | [90,100] |
| --- | --- | --- | --- | --- | --- | --- | --- | --- | --- | --- | --- |
| **FN genes** | <10^-3^ | 0.99 | 1 | 0.98 | 1 | 1 | 0.97 | 0.08 | 0.96 | <10^-3^ | <10^-3^ |
| **Total genes** | 0.36 | 0.52 | 0.4 | 0.62 | 0.57 | 0.65 | 0.8 | 0.69 | 0.17 | 0.67 | 0.29 |
