# Supplementary material for: A Statistical Framework for Improving Genomic Annotations of Prokaryotic Essential Genes
Source: PLoS One. 2013 Mar 8;8(3):e58178. doi: 10.1371/journal.pone.0058178 (PMC3592911; doi:10.1371/journal.pone.0058178)
Supplement: Table S3 — (DOC) [file pone.0058178.s005.doc]

**Table S3**. Bacterial strains and plasmids used in this study.

| **Strain or plasmid** | **Genotype or description** | **Ref. or source** |
| --- | --- | --- |
| ***P. aeruginosa*** |  |  |
| PAO1 | Wild type | Our collection |
| PAO1Δ0985::Gm | Gm ^r^; PA0985::Gm | This study |
| PAO1Δ2954::Gm | Gm ^r^; PA2954::Gm | This study |
| PAO1Δ2143::Gm | Gm ^r^; PA2143::Gm | This study |
| ***E. coli*** |  |  |
| DH5α | F^-^ Φ80d*lacZ*Δ*M15 endA1 recA1 hsdR17(r_K_ - m_K_ -)*  *supE44 thi-1 gyrA96* Δ *(lacZYA-argF)*U169 |  |
| S17-1 | Pro – Res – Mod ^+^ *recA*; integrated RP4-Tet::Mu-Kan::Tn*7*, Mob ^+^ |  |
| **Plasmids** |  |  |
| pBluescript SK+ | Cloning vector | Stratagene |
| pSKΔ2143 | Two 1 kb flanking fragments of PA2143 were ligated and cloned into pBluescript SK+ | This study |
| pUCGm | Source of 931-bp gentamicin resistance cassette |  |
| pSKΔ2143::Gm | Gm^r^  cassette inserted into pSKΔ2143 | This study |
| pBBR1MCS-3 | Broad-host-range vector |  |
| pUCP20 | pUC18/19-derived shuttle vector |  |
| pSU20 | A derivative of pUCP20; Tet^r^ | This study |
| pEX100T | *oriT mob sacB* gene replacement vector |  |
| pEX100TΔ0985::Gm | pEX100T carrying a PA0985::Gm gene replacement construct | This study |
| pEX100TΔ2954::Gm | pEX100T carrying a PA2954::Gm gene replacement construct | This study |
| pEX100TΔ2143::Gm | pEX100T carrying a PA2143::Gm gene replacement construct | This study |
| pEX100TΔ0723::Gm | pEX100T carrying a PA0723::Gm gene replacement construct | This study |
| pEX100TΔ3746::Gm | pEX100T carrying a PA3746::Gm gene replacement construct | This study |
| pEX100TΔ4260::Gm | pEX100T carrying a PA4260::Gm gene replacement construct | This study |
| pEX100TΔ4238::Gm | pEX100T carrying a PA4238::Gm gene replacement construct | This study |
| pSU0723 | pSU20 with PA0723 | This study |
| pSU3746 | pSU20 with PA3746 | This study |
| pSU4260 | pSU20 with PA4260 | This study |
| pSU4238 | pSU20 with PA4238 | This study |
