# Supplementary material for: A Statistical Framework for Improving Genomic Annotations of Prokaryotic Essential Genes
Source: PLoS One. 2013 Mar 8;8(3):e58178. doi: 10.1371/journal.pone.0058178 (PMC3592911; doi:10.1371/journal.pone.0058178)
Supplement: Table S4 — (DOC) [file pone.0058178.s006.doc]

**Table S4.** Comparison of performance of different types of computational methods for predicting essential genes

| **Method Type** | **Method** | **Correct Predicted Essential / Total Predicted Essential** | **Sensitivity** | **Specificity** | **Precision** | **Target**  **Organism** |
| --- | --- | --- | --- | --- | --- | --- |
| Unsupervised approach | Our model | 181/492 | 0.60 | 0.91 | 37% | *E. coli* |
|  | Gerdes 2003; | 205/620 | 0.68 | 0.88 | 33% | *E. coli* |
| Homology mapping | Deng 2011; | 195/560 | 0.65 | 0.89 | 35% | *E. coli* |
| Constraint-based methods | Joyce 2006; | 77/182 | 0.26 | 0.25 | 42% | *E. coli* |
| Supervised machine learning | Deng 2011; | 218/492 | 0.73 | 0.92 | 44% | *E. coli* |
|  | Gustafson 2006; | 156/295 | 0.52 | 0.96 | 53% | *E. coli* |
