# Supplementary material for: A Statistical Framework for Improving Genomic Annotations of Prokaryotic Essential Genes
Source: PLoS One. 2013 Mar 8;8(3):e58178. doi: 10.1371/journal.pone.0058178 (PMC3592911; doi:10.1371/journal.pone.0058178)
Supplement: Text S1 — (DOC) [file pone.0058178.s007.doc]

**RESULTS**

**1. Experimental results from allelic exchange experiments in *P. aeruginosa* strain *PAO1*.**

**PA0985, PA2954 and PA2143 are non-essential genes.** Many trans**-**conjugates appeared after biparental mating on Gm-containing LB agar. Some were carbenicillin-resistant (Cbr) and others Carbenicillin-sensitive (Cbs). We randomly selected two Cbr and Cbs colonies and isolated their genomic DNA. PCR with primers flanking the target genes was used to assess the allelic exchange of the mutant gene for the wild-type allele. As shown in **Fig. S2**, parental *PAO1* yielded a specific PCR product of the 1,497 bp wild-type gene PA0985, while two Cbr conjugates (#1 and #2) gave two bands, of which the size of larger band was equal to the length of mutant copy of PA0985 (1,496 bp + 931 bp of Gmr cassette insertion =2,428 bp in length) and the bottom band was an amplification product of the wild-type copy of PA0985, indicating the two Cbr conjugates were single cross-over (merodiploid) recombinants. In comparison, two Cbs conjugates (#3 and #4) yielded only one band of the 2,428 bp mutant copy of PA0985, suggesting they were double cross-over recombinants, of which the pEX100T vector backbone was lost. PA2954 and PA2143 were mutated in the same fashion as PA0985.

**PA0723, PA3746, PA4260 and PA4238 are essential genes.** When we attempted to construct isogenic mutants of the PA0723 (*coaB*, coat protein B of bacteriophage Pf1), PA3746 (*ffh*, signal recognition particle protein Ffh, PA4260 (*rplB*, 50S ribosomal protein L2) and PA4238 (*rpoA*, sigma factor RpoA) genes, we had great difficulty to obtain homologous recombinants, even as merodiploids. For mutagenesis of PA0723 and PA3746, all conjugates arising on gentamicincontaining LB plates were resistant to carbenicillin, and all colonies that endured sucrose counter-selection were still Cbr. The same situation occurred even when we provided an extra plasmid copy of each gene as pSU0723 or pSU3746. PCR analysis indicated that all Cbr colonies were diploid recombinants. For inactivation of PA4260 and PA4238, we tried several times and failed to observe any Gmr trans-conjugates unless a wild-type copy of PA4260- or PA4238-carrying plasmids, pSU4260 or pSU4238, were provided *in trans*. All colonies arising from LB plates containing 7% sucrose and gentamicin were resistant to carbenicillin and diploid recombinants analyzed by PCR.

**MATERIALS AND METHODS**

**1. Experimental validation in *P. aeruginosa PAO1***

**Bacterial strains, media, and growth conditions.** The bacterial strains and the plasmids used in this study are listed in **Table S2**. *P. aeruginosa* strains were routinely grown aerobically at 37°C in Luria-Bertani (LB) broth, or on LB agar. Antibiotics were added at the following concentrations in μg/ml when required: for *P. aeruginosa* strains, gentamicin, 150; tetracycline, 100; and carbenicillin, 200.

**DNA manipulations.** Genomic DNA isolation, PCR, restriction enzyme digestion, ligation, cloning and DNA electrophoresis were performed according to standard techniques . All oligonucleotide primers were synthesized by Integrated DNA Technologies (IDT). PCR was performed using either Choice *Taq* Mastermix (Denville Scientific Inc.) or *Pfu* DNA polymerase (Strategene). Plasmid DNA was purified using a QIAprep Spin miniprep kit (QIAGEN) as recommended by the manufacturer. DNA fragments in gels were purified using either a QIAquick PCR purification kit (QIAGEN) or a QIAquick gel extraction kit (QIAGEN). All cloned inserts were confirmed by automated DNA sequencing performed at the DNA Core Facility of the Cincinnati Children’s Hospital Medical Center. Plasmids were introduced into *E.* *coli* by CaCl2-mediated transformation and into *P. aeruginosa* strains by either electroporation or

biparental mating.

***In vitro* mutagenesis and gene replacement.** The strategy for mutagenesis of the *P. aeruginosa* genes was facilitated by insertional mutagenesis with a 931-bp gentamicin resistance (Gmr) cassette from pUCGM , and the gene replacement vector pEX100T harboring the *sacB* gene as a counter-selectable marker as described by Schweizer . The *aacC1* gene within the Gmr cassette was inserted in the same orientation of the target gene to avoid possible polar effect on downstream loci. All fragments cloned into pEX100T harbored 1 kb of flanking sequence of the target gene. To mutagenize the PA0985 gene, a 3,497 bp *Sal*I-*Bam*HI PA0985-containing fragment was amplified by PCR with primers U0985/Sal5′ and D0985/Bm3′ and cloned into pEX100T to form pEX0985. A Gmr cassette was inserted into the unique *Pst*I site of PA0985 in pEX0985. The resulting plasmid, pEXΔ0985::Gm, was introduced into the mobilizing *E. coli* strain S17-1, and S17-1 (pEXΔ0985::Gm) was conjugated with *P. aeruginosa* *PAO1* by filter mating. After incubation for 24 h at 37°C, the cells were suspended into 1 ml of 0.9% NaCl and plated on LB-Gm plates. Colonies arising after 48 h were purified on the same medium and cultured in LB broth. The stationary phase culture was serially diluted and plated onto LB agar plates containing gentamicin and 7% sucrose. Gm and sucrose-resistant colonies were tested for sensitivity to carbenicillin to confirm the lost of the vector backbone. Gene replacement was confirmed by PCR amplification of the chromosomal DNA with primers PA0985/Sm5′ and PA0985/Hind3′ flanking the PA0985. Similar cloning strategy was used to generate plasmid constructs pEXΔ2954::Gm, pEXΔ3746::Gm, pEXΔ4260::Gm, and pEXΔ4238::Gm for inactivation of PA2954, PA3746, PA4260 and PA4238, respectively. To mutagenize the PA2143 gene, a 1 kb *Eco*RI-*Hind*III PCR fragment upstream of PA2143 was ligated with a 1 kb *Hind*III-*Eco*RI PCR fragment downstream of PA2143 and cloned into the *Eco*RI site of pBluescript SK+ to form pSKΔ2143. A Gmr cassette was excised from pUCGM and inserted into the *Hind*III site of pSKΔ2143. The resulting plasmid pSKΔ2143::Gm was digested with *Eco*RI, and the fragment harboring the Gmr cassette was cloned into pEX100T to form pEXΔ2143::Gm. Similar cloning strategy was used to create pEXΔ0723::Gm for the mutagenesis of PA0723. Considering that some genes are known to be essential, a complementing plasmid carrying a wild-type copy of the target gene was also constructed as follows: a 1.6 kb tetracycline resistance cassette from pBBR1MCS-3 was excised by *Hind*III, made blunt by T4 DNA polymerase and inserted into the unique *Sca*I site within the *bla* gene in pUCP20 , creating plasmid pSU20. The 249 bp PA0723 gene, an 822 bp PA4260 gene and a 1,374 bp PA3746 genes were amplified by PCR and cloned between *Eco*RI and *Xba*I sites of pSU20 to form pSU0723, pSU4260 and pSU3746, respectively. A 1,002 bp PA4238 gene was cloned between *Eco*RI and *Hind*III sites of pSU20, creating pSU4238.

**FIGURE LEGENDS**

**Figure S1**. Identification of essential genes by TM.

After randomly inserting transposons into a bacterial genome, the surviving colonies are selected and sequenced. If a transposon is never detected in a gene (A and D), it is determined as essential because any insertion in this gene would have caused the bacteria to die and thus unobservable.

However, some genes (D), especially short ones, are simply missed by chance. This will result in false essential assignments where D is mistakenly determined as essential. Likewise, if a transposon is detected in a gene (B and C), it is determined as non-essential because the bacteria survive without normal function of this gene product. However, sometimes a transposon insertion does not completely disrupt a gene’s function (B), e.g., insertions in the extreme ends (5’ or 3’ ends) of a gene. This can result in false non-essential assignments where B is mistakenly determined as non-essential genes.

**Figure S2**. PCR analysis of PA0985 merodiploid (single crossover) or mutant (double crossover) recombinant strains. WT, wild-type 1,497 bp PCR product of the PA0985 gene; 1 and 2, two merodiploid strains harboring wild-type and mutant copies; 3 and 4, mutant strains.

**REFERENCES**

1. Sambrook J, Maniatis T, Fritsch EF (1989) Molecular cloning : a laboratory manual. Cold Spring Harbor, N.Y.: Cold Spring Harbor Laboratory Press. v. (various pagings) p.

2. Schweizer HD (1993) Small broad-host-range gentamycin resistance gene cassettes for site-specific insertion and deletion mutagenesis. Biotechniques 15: 831-834.

3. Schweizer HP, Hoang TT (1995) An improved system for gene replaceent and xylE fusion analysis in Pseudomonas aeruginosa. Gene 158: 15-22.

4. Schweizer HP (1992) Allelic exchange in Pseudomonas aeruginosa using novel ColE1-type vectors and a family of cassettes containing a portable oriT and the counter-selectable Bacillus subtilis sacB marker. Mol Microbiol 6: 1195-1204.

5. Hutchison CA, Peterson SN, Gill SR, Cline RT, White O, et al. (1999) Global transposon mutagenesis and a minimal Mycoplasma genome. Science 286: 2165-2169.

6. Hare RS, Walker SS, Dorman TE, Greene JR, Guzman LM, et al. (2001) Genetic footprinting in bacteria. J Bacteriol 183: 1694-1706.

7. Akerley BJ, Rubin EJ, Novick VL, Amaya K, Judson N, et al. (2002) A genome-scale analysis for identification of genes required for growth or survival of Haemophilus influenzae. Proc Natl Acad Sci U S A 99: 966-971.

8. Gerdes SY, Scholle MD, Campbell JW, Balazsi G, Ravasz E, et al. (2003) Experimental determination and system level analysis of essential genes in Escherichia coli MG1655. J Bacteriol 185: 5673-5684.

9. Sassetti CM, Boyd DH, Rubin EJ (2003) Genes required for mycobacterial growth defined by high density mutagenesis. Mol Microbiol 48: 77-84.

10. Jacobs MA, Alwood A, Thaipisuttikul I, Spencer D, Haugen E, et al. (2003) Comprehensive transposon mutant library of Pseudomonas aeruginosa. Proc Natl Acad Sci U S A 100: 14339-14344.

11. Tong X, Campbell JW, Balazsi G, Kay KA, Wanner BL, et al. (2004) Genome-scale identification of conditionally essential genes in E. coli by DNA microarrays. Biochem Biophys Res Commun 322: 347-354.

12. Salama NR, Shepherd B, Falkow S (2004) Global transposon mutagenesis and essential gene analysis of Helicobacter pylori. J Bacteriol 186: 7926-7935.

13. Glass JI, Assad-Garcia N, Alperovich N, Yooseph S, Lewis MR, et al. (2006) Essential genes of a minimal bacterium. Proc Natl Acad Sci U S A 103: 425-430.

14. Liberati NT, Urbach JM, Miyata S, Lee DG, Drenkard E, et al. (2006) An ordered, nonredundant library of Pseudomonas aeruginosa strain PA14 transposon insertion mutants. Proc Natl Acad Sci U S A 103: 2833-2838.

15. Suzuki N, Okai N, Nonaka H, Tsuge Y, Inui M, et al. (2006) High-throughput transposon mutagenesis of Corynebacterium glutamicum and construction of a single-gene disruptant mutant library. Appl Environ Microbiol 72: 3750-3755.

16. Filiatrault MJ, Picardo KF, Ngai H, Passador L, Iglewski BH (2006) Identification of Pseudomonas aeruginosa genes involved in virulence and anaerobic growth. Infect Immun 74: 4237-4245.

17. Gallagher LA, Ramage E, Jacobs MA, Kaul R, Brittnacher M, et al. (2007) A comprehensive transposon mutant library of Francisella novicida, a bioweapon surrogate. Proc Natl Acad Sci U S A 104: 1009-1014.

18. French CT, Lao P, Loraine AE, Matthews BT, Yu H, et al. (2008) Large-scale transposon mutagenesis of Mycoplasma pulmonis. Mol Microbiol 69: 67-76.

19. Cameron DE, Urbach JM, Mekalanos JJ (2008) A defined transposon mutant library and its use in identifying motility genes in Vibrio cholerae. Proc Natl Acad Sci U S A 105: 8736-8741.

20. Langridge GC, Phan MD, Turner DJ, Perkins TT, Parts L, et al. (2009) Simultaneous assay of every Salmonella Typhi gene using one million transposon mutants. Genome Res 19: 2308-2316.

21. Murray GL, Morel V, Cerqueira GM, Croda J, Srikram A, et al. (2009) Genome-wide transposon mutagenesis in pathogenic Leptospira species. Infect Immun 77: 810-816.

22. Chaudhuri RR, Allen AG, Owen PJ, Shalom G, Stone K, et al. (2009) Comprehensive identification of essential Staphylococcus aureus genes using Transposon-Mediated Differential Hybridisation (TMDH). BMC Genomics 10: 291.

23. Molina-Henares MA, de la Torre J, Garcia-Salamanca A, Molina-Henares AJ, Herrera MC, et al. (2010) Identification of conditionally essential genes for growth of Pseudomonas putida KT2440 on minimal medium through the screening of a genome-wide mutant library. Environ Microbiol 12: 1468-1485.

24. Lamichhane G, Freundlich JS, Ekins S, Wickramaratne N, Nolan ST, et al. (2011) Essential metabolites of Mycobacterium tuberculosis and their mimics. MBio 2: e00301-00310.

25. Christen B, Abeliuk E, Collier JM, Kalogeraki VS, Passarelli B, et al. (2011) The essential genome of a bacterium. Mol Syst Biol 7: 528.

26. Soemphol W, Deeraksa A, Matsutani M, Yakushi T, Toyama H, et al. (2011) Global analysis of the genes involved in the thermotolerance mechanism of thermotolerant Acetobacter tropicalis SKU1100. Biosci Biotechnol Biochem 75: 1921-1928.

27. Mendum TA, Newcombe J, Mannan AA, Kierzek AM, McFadden J (2011) Interrogation of global mutagenesis data with a genome scale model of Neisseria meningitidis to assess gene fitness in vitro and in sera. Genome Biol 12: R127.

28. Stahl M, Stintzi A (2011) Identification of essential genes in C. jejuni genome highlights hyper-variable plasticity regions. Funct Integr Genomics 11: 241-257.

29. Simon R, Priefer UB, Puhler A (1983) A broad host range mobilization system for in vivo genetic engineering: transposon mutagenesis in Gram negative bacteria. Nature Biotechnology 1: 784-791.

30. Kovach ME, Elzer PH, Hill DS, Robertson GT, Farris MA, et al. (1995) Four new derivatives of the broad-host-range cloning vector pBBR1MCS, carrying different antibiotic-resistance cassettes. Gene 166: 175-176.

31. West SE, Schweizer HP, Dall C, Sample AK, Runyen-Janecky LJ (1994) Construction of improved Escherichia-Pseudomonas shuttle vectors derived from pUC18/19 and sequence of the region required for their replication in Pseudomonas aeruginosa. Gene 148: 81-86.

32. Deng J, Deng L, Su S, Zhang M, Lin X, et al. (2011) Investigating the predictability of essential genes across distantly related organisms using an integrative approach. Nucleic Acids Res 39: 795-807.

33. Joyce AR, Reed JL, White A, Edwards R, Osterman A, et al. (2006) Experimental and computational assessment of conditionally essential genes in Escherichia coli. J Bacteriol 188: 8259-8271.

34. Gustafson AM, Snitkin ES, Parker SC, DeLisi C, Kasif S (2006) Towards the identification of essential genes using targeted genome sequencing and comparative analysis. BMC Genomics 7: 265.
